# Modulation of Syndecan-1 Shedding after Hemorrhagic Shock and Resuscitation

**DOI:** 10.1371/journal.pone.0023530

**Published:** 2011-08-19

**Authors:** Ricky J. Haywood-Watson, John B. Holcomb, Ernest A. Gonzalez, Zhanglong Peng, Shibani Pati, Pyong Woo Park, WeiWei Wang, Ana Maria Zaske, Tyler Menge, Rosemary A. Kozar

**Affiliations:** 1 Department of Surgery, The University of Texas Health Science Center at Houston, Houston, Texas, United States of America; 2 Center for Translational Injury and Research (CeTIR), The University of Texas Health Science Center at Houston, Houston, Texas, United States of America; 3 Department of Surgery, University of Texas Southwestern Austin, Austin, Texas, United States of America; 4 Division of Respiratory Diseases, Children's Hospital, Harvard Medical School, Boston, Massachusetts, United States of America; 5 Center for Clinical and Translational Sciences, The University of Texas Health Science Center at Houston, Houston, Texas, United States of America; Medical College of Georgia, United States of America

## Abstract

The early use of fresh frozen plasma as a resuscitative agent after hemorrhagic shock has been associated with improved survival, but the mechanism of protection is unknown. Hemorrhagic shock causes endothelial cell dysfunction and we hypothesized that fresh frozen plasma would restore endothelial integrity and reduce syndecan-1 shedding after hemorrhagic shock. A prospective, observational study in severely injured patients in hemorrhagic shock demonstrated significantly elevated levels of syndecan-1 (554±93 ng/ml) after injury, which decreased with resuscitation (187±36 ng/ml) but was elevated compared to normal donors (27±1 ng/ml). Three pro-inflammatory cytokines, interferon-γ, fractalkine, and interleukin-1β, negatively correlated while one anti-inflammatory cytokine, IL-10, positively correlated with shed syndecan-1. These cytokines all play an important role in maintaining endothelial integrity. An *in vitro* model of endothelial injury then specifically examined endothelial permeability after treatment with fresh frozen plasma orlactated Ringers. Shock or endothelial injury disrupted junctional integrity and increased permeability, which was improved with fresh frozen plasma, but not lactated Ringers. Changes in endothelial cell permeability correlated with syndecan-1 shedding. These data suggest that plasma based resuscitation preserved endothelial syndecan-1 and maintained endothelial integrity, and may help to explain the protective effects of fresh frozen plasma after hemorrhagic shock.

## Introduction

Hemorrhagic shock is the most common cause of potentially preventable death after both civilian and combat traumatic injury [1]. Despite the significant effort expended on mechanistic resuscitation studies, several large randomized multicenter clinical trials have unfortunately failed to demonstrate any clinically significant outcome differences [2]–[4]. Recently, data from both military [5], [6] and civilian studies [7]–[9] have associated survival benefit following massive transfusion (>10 units packed red cells in 24 hours) with the implementation of a high ratio fresh frozen plasma (FFP) to red cell resuscitation strategy. This change in resuscitation centers around the early and increased use of plasma and platelets and decreased crystalloid utilization. These changes have been associated with a significant increase in early survival, though the studies are retrospective and the mechanism of protection is unknown. To begin to investigate the molecular pathways responsible for protection by FFP-based resuscitation, we are focusing on the role of the endothelial cell in maintaining endothelial integrity [10]. Endothelial dysfunction and hyperpermeability have been implicated in the morbidity and mortality associated with sepsis, organ failure and hemorrhagic shock [11]–[13].

The glycocalyx is a network of soluble plasma components that projects from the endothelial cell surface and plays a key role in maintaining endothelial integrity [14]. It consists of proteoglycans and glycoproteins attached to the cell surface. Cell adhesion molecules constitute several of the glycoproteins. With injury to the glycocalyx, adhesion molecules are exposed, allowing pathologic neutrophil-endothelial cell interactions. Other glycoproteins within the glycocalyx are important to coagulation, fibrinolysis, and hemostasis [15]. The major cell surface proteoglycan is syndecan, whose extracellular domain is substituted with heparan sulfate chains and promotes interaction with plasma proteins [16]. There are four members (syndecan 1–4) that comprise the syndecan family. While syndecan-1 is found primarily on epithelial cells, recent data suggests that it also found on endothelial cells and plays an important role in endothelial cell function after hemorrhagic shock [17], [18]. We therefore hypothesized that hemorrhagic shock would disrupt endothelial integrity by promoting syndecan-1 shedding from the endothelial cell surface and that shed syndecan-1 would be lessened by plasma based resuscitation in severely injured patients in hemorrhagic shock.

Cytokines are significant mediators in the systemic and local inflammatory response observed in critically ill and injured patients [19], [20]. Studies have shown that cytokines recruit neutrophils into the vasculature that then traverse the injured endothelium and cause end organ damage [21]. The many roles that cytokines play in the pathophysiology of endothelial damage are still unclear and to our knowledge, no reports have identified a relationship between cytokines and markers of endothelial injury after hemorrhagic shock. We therefore also hypothesized that patients presenting in hemorrhagic shock would have temporally increased shedding of syndecan-1, which would correlate with increased production of inflammatory cytokines. We identified four cytokines that correlated with syndecan shedding then used them in an *in vitro* model of endothelial injury to examine FFP's effect on endothelial integrity.

## Results

### Human Study

#### Severely injured patients

A total of 32 patients were enrolled in this pilot study. Patient demographics, injury severity, parameters of shock, and pre-intensive care unit (ICU) resuscitation are depicted in Table 1. This severely injured cohort (Injury Severity Scale [ISS], 31±2) had an overall mortality of 44% (14/32). Sixteen causes of death in 14 patients included: head injury [7 (50%)], hemorrhage [5, (36%)], withdrawal of care [2 (14%)], cardiac arrest [1 (7%)], and multiple organ failure (MOF) [1 (7%)]; two patients had mortality attributed to both head injury and hemorrhage. Time to death was early, consistent with severe injury: ten patients died <24 hours from arrival, one at 48 hours, one at 72 hours, and one at day four. There was only one late death (>30 days from MOF). Multiple organ failure occurred in 3 of the 21 patients (14%) that survived over 48 hours.

**Table 1:** Shock Resuscitation Cohort.

Parameter	Value
Sample size	32
Age (yr)	40±3
Men (n [%])	24 (75)
ISS	31±2
Blunt mech (n [%])	26 (81)
ED INR	1.9±0.2
ED BD (mEq/L)	10±1
ED HgB (g/dL)	11.7±0.4
ED Temp (°F)	96.9±0.2
Pre-ICU crys (L)	3.7±0.4
Pre-ICU pRBC (unit)	6±1
Pre-ICU FFP (unit)	7±2
Pre-ICU platelets (L)	0.3±0.05
ICU INR	1.6±0.07
ICU BD (mEq/L)	4±1
ICU Temp (°F)	96.5±0.4
ICU LOS (days)	9±2

ISS, injury severity score; Blunt mech, blunt mechanism of injury; ED INR, international normalized ratio in emergency department at hospital admission; ED BD, base deficit in emergency department at hospital admission; ED HgB, hemoglobin in emergency department at hospital admission; ED Temp, body core temperature in emergency department at hospital admission; pre-ICU crys, crystalloid fluid volume infused from hospital to ICU admission; pre-ICU PRBC, packed red blood cell volume infused from hospital to ICU admission; pre-ICU FFP, fresh frozen plasma volume infused from hospital to ICU admission; pre-ICU platelets, platelet volume infused from hospital to ICU admission; ICU INR, INR at ICU admission; ICU BD, base deficit in ICU at admission; ICU Temp, body core temperature in ICU at admission; ICU LOS, ICU length of stay.

#### Injured patients in shock shed syndecan-1

**Figure 1:**
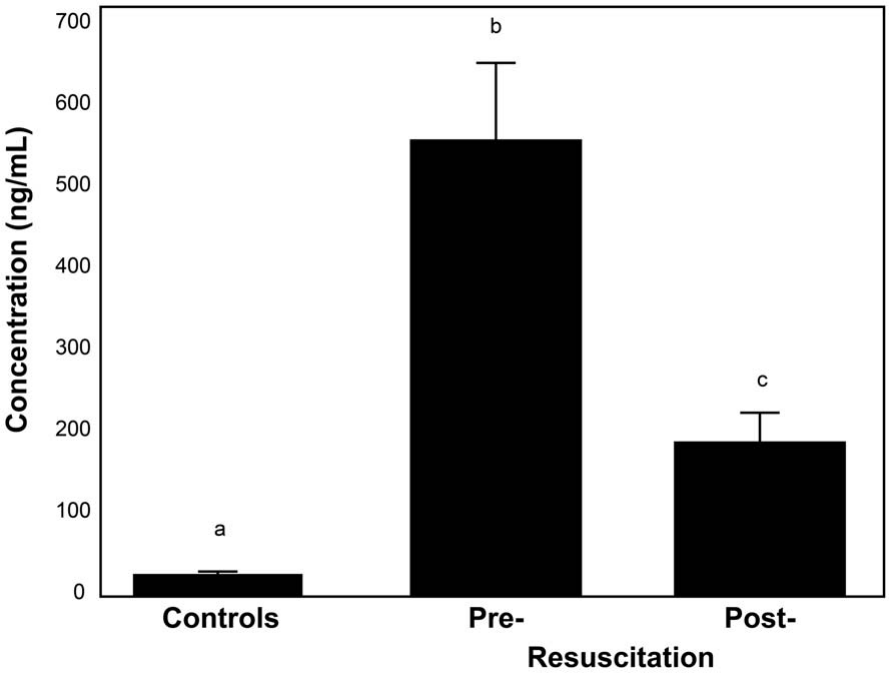
Syndecan-1 is shed following hemorrhagic shock. Plasma syndecan-1 was measured in patients presenting in hemorrhagic shock. The mean plasma concentration is shown for controls, pre-resuscitation, and post-resuscitation time points. Syndecan-1 concentrations were markedly elevated pre-resuscitation and remained significantly elevated post-resuscitation compared to controls. Resuscitated patients reached the ICU an average of 7±0.75 hours after arrival in the emergency room. Results are presented as mean ± SEM, means notated with letters indicate statistical differences between groups.

Injured patients in shock demonstrated markedly elevated plasma syndecan-1 levels (554±93 ng/ml) upon arrival to the emergency department, with seven patients having levels greater than 1000 ng/ml (Figure 1). Levels significantly decreased with resuscitation (187±36 ng/ml, p = 0.001) but remained elevated above that of controls (27 ng/ml±1; p<0.001). Post resuscitation syndecan levels in patients who survived were 144±141 ng/ml while in nonsurvivors were 289±226 ng/ml , p = 0.15.

#### Syndecan-1 correlated with specific inflammatory cytokines

**Figure 2:**
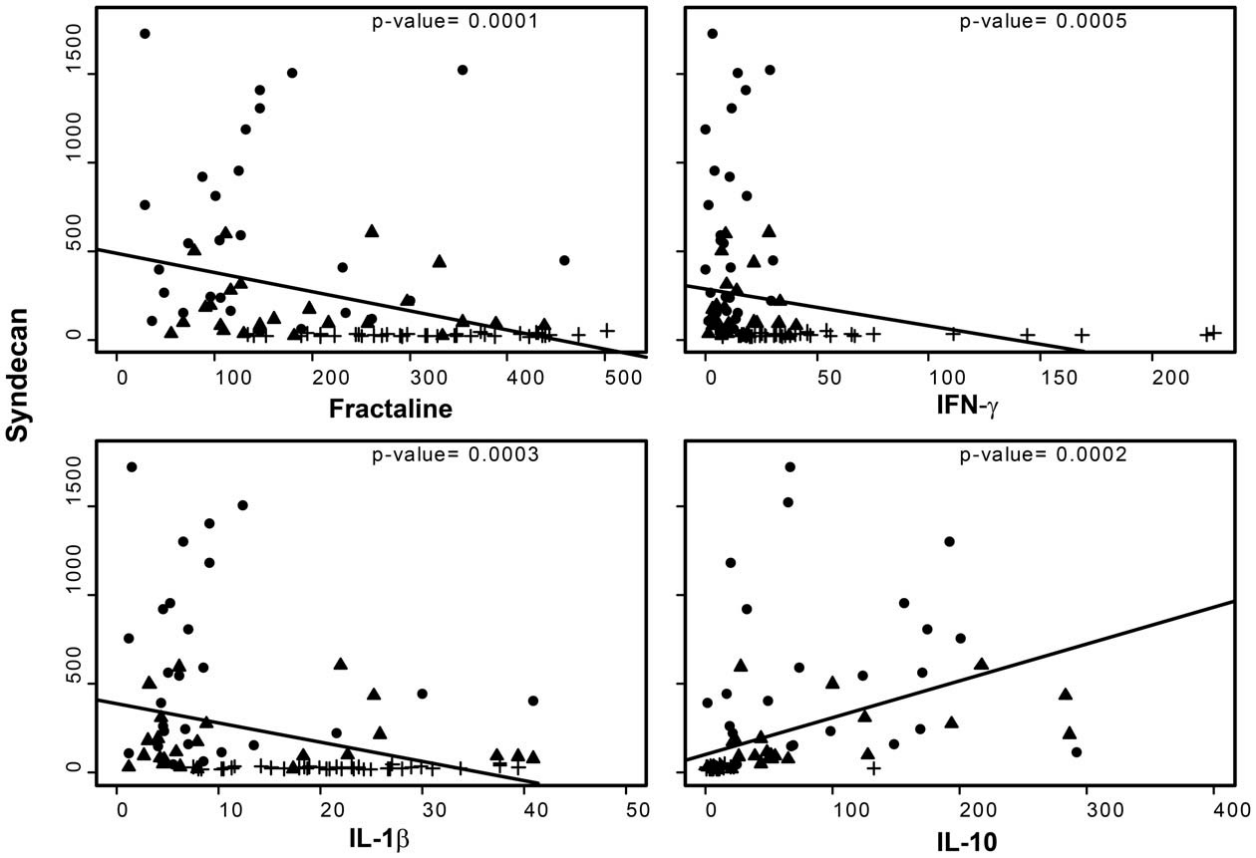
Cytokines correlate with syndecan-1 shedding. Scatter plots, with linear regression lines, of the cytokines identified to correlate with syndecan-1 are shown. Fractalkine, IFN-γ, and IL-1β were negatively correlated with syndecan-1. IL-10, on the other hand was positively correlated. Symbols: + normal donors, • pre-resuscitation, ▴post-resuscitation.

Shock patients demonstrated a significant increase in the expression of inflammatory cytokines (Table S1). Due to the temporal relationship of these findings we explored a correlation between changes in shed syndecan-1 and cytokine expression. Of the 39 cytokines we measured, four cytokines were identified that correlated with shed syndecan-1: IFN-γ, fractalkine, and IL-1β were negatively correlated while IL-10 was positively correlated (Figure 2 and Table 2).

**Table 2:** Correlation of syndecan-1 to cytokines.

	beta (se)^a^	p-value	#OOR	R^2^
Fractalkine	−1.08(0.28)	0.0001^b^	0	0.12
IFNγ	−2.08(0.6)	0.0005^b^	1	0.36
IL-1β	−10.79(2.95)	0.0003^b^	0	0.10
IL-10	2.08(0.55)	0.0002^b^	0	0.17

se = standard error of beta estimate; OOR = out of range.

a : beta = change of syndecan-1 with 1 unit increase of cytokine.

b : statistically significant with Bonferroni correction (p-value<0.05/38).

### *In vitro* model of endothelial injury

The association of these four cytokines with syndecan-1 shedding after hemorrhagic shock is a novel finding. We therefore incorporated IL-1β into an *in vitro* model of endothelail injury using hypoxia/reoxygenation in human umbilical vascular endothelial cells (HUVECs) to specifically study endothelial integrity. IL-1β was added at the time of shock as a pro-inflammatory cytokine associated with syndecan-1 shedding. Endothelial integrity was compared between lactated Ringers (LR), the standard crystalloid used in shock resuscitation, and fresh frozen plasma. As all of our patients received both LR and plasma (Table 1) during resuscitation, we evaluated their individual effects on vascular integrity.

#### Vascular integrity is disrupted by shock but mitigated by FFP

**Figure 3:**
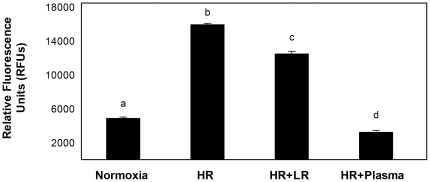
Endothelial cell permeability is decreased by FFP in an in vitro model of endothelial injury. HUVEC's were stimulated with IL-1β and subjected to hypoxia for four hours followed by rexoygenation and incubation for an additional four hours in media alone (hypoxia/reoxygenation, HR), 5% lactated Ringers (LR), or 5% FFP, and compared to normoxia. *In vitro* permeability was assessed by fluoresceine-isothiocyanate [FITC] labeled dextran transport. Permeability was significantly increased by HR compared to normoxia, lessened by LR, but FFP decreased permeability to below that of normoxia. Results are presented as means ± SEM, n = 6/group. Means notated with letters indicate statistical differences between groups.

Diverse pathologic conditions such as sepsis, cancer, and hemorrhagic shock, destabilize the intact endothelium, resulting in hyperpermeability. *In vitro* permeability after endothelial injury (15,916±206 RFU's) was significantly increased compared to normoxic controls (4898±157 RFU's) and lessened by LR (12,477±370 RFUs). Consistent with our previous data, FFP (3268±218RFU) decreased hyperpermeability to below that of normoxic controls (Figure 3) [10].

**Figure 4:**
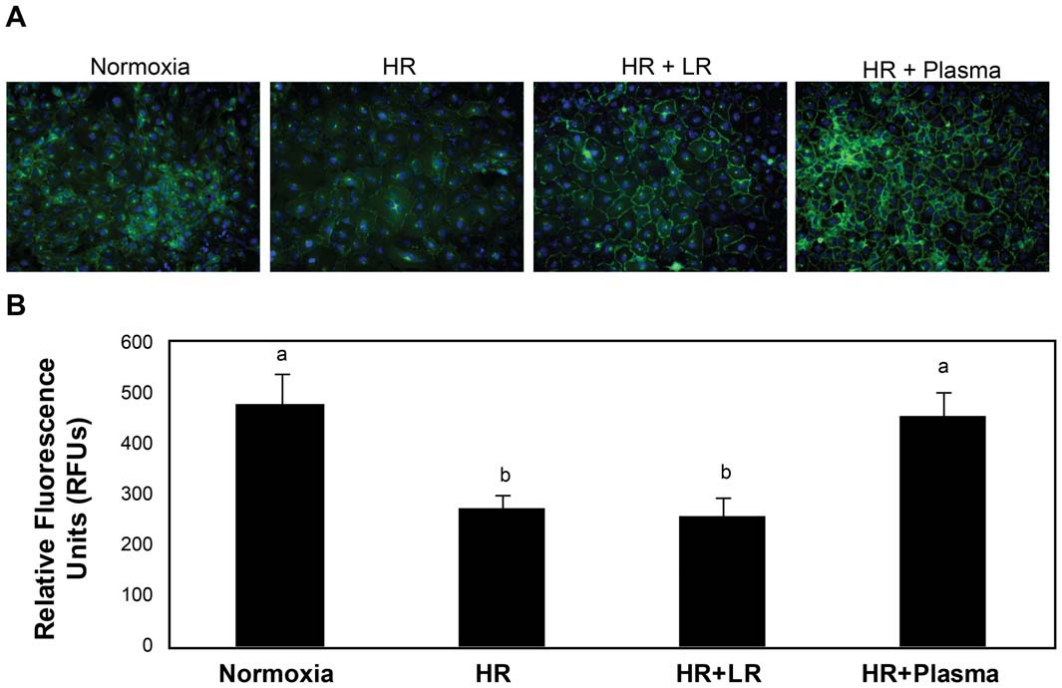
VE-cadherin immunoreactivity is enhanced by FFP in an in vitro model of endothelial injury. HUVEC's were stimulated with IL-1β and subjected to hypoxia for four hours followed by rexoygenation and incubation for an additional four hours in media alone (hypoxia/reoxygenation, HR), 5% lactated Ringers (LR), or 5% FFP, and compared to normoxia. A. Cells were labeled with antibody to VE-cadherin and images captured with an IX71 inverted microscope. Original image magnification, ×40. B. The relative fluorescence intensity was quantified using Image J software (NIH) and reported as relative fluorescence units (RFU). Results are presented as means ± SEM, n = 3/group. VE-cadherin immunoreactivity was reduced by HR and LR compared to normoxia, but enhanced by FFP.

**Figure 5:**
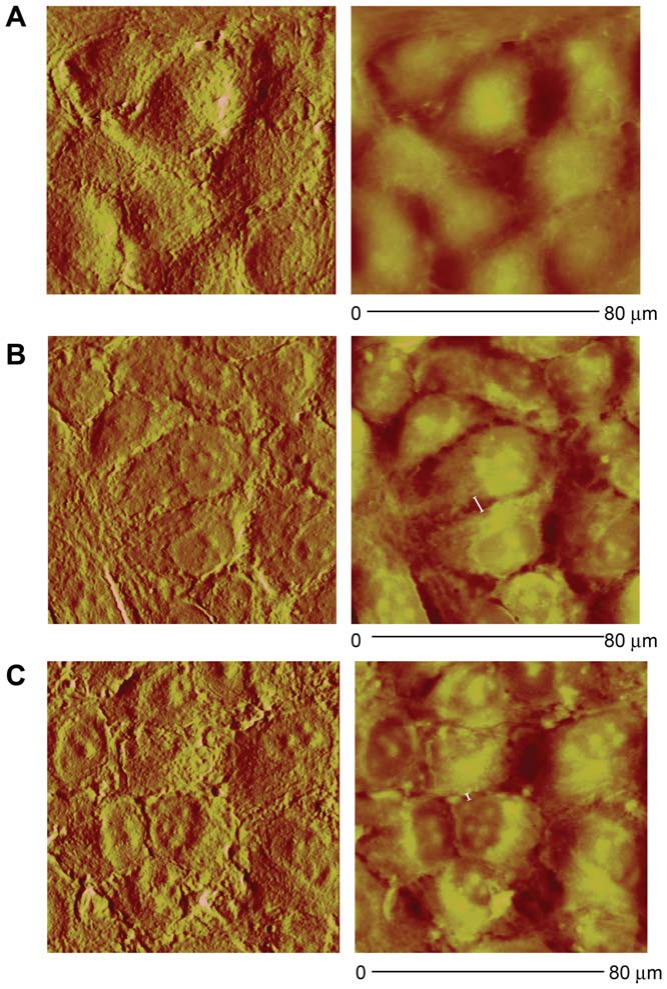
Endothelial surface ultrastructure is restored by FFP in an in vitro model of shock. HUVEC's were cultured under normoxic conditions or stimulated with IL-1β and subjected to hypoxia for four hours followed by rexoygenation and incubation for an additional four hours in 5% lactated Ringers (LR), or 5% FFP and compared to normoxia. Cell surface ultrastructure was assessed using atomic force microscopy, 80 µm area scanned for all images. The deflection (left) and corresponding height (right) topographic images are shown from representative images of three separate experiments for each group. Normoxic controls (A) demonstrate no gaps between cells whereas areas of thinning between cell junctions are seen after LR (B) but reduced by FFP (C). Gaps are indicated by white lines.

Endothelial integrity is compromised when junctional proteins and adherens junction protein vascular endothelial cadherin (VE-cadherin) interactions are disrupted [22]. VE-cadherin immunoreactivity was reduced after injury (274±26 RFU) and LR (257±38 RFU) compared to normoxic controls (480±60 RFU), but was enhanced by FFP (457±46 RFU) (Figure 4 A and B). To further examine endothelial cell junctional integrity by resuscitative agents, surface ultrastructure was assessed using atomic force microscopy (AFM) (Figure 5). As demonstrated by color enhanced 3-D topographic rendering, normoxic cells had no demonstrable gaps detected whereas large gaps between cell were seen after endothelial injury in the LR group (6.48±1.11 µm, p = 0.004 vs normoxic controls) and were significantly lessened by FFP (2.38±0.25 µm, p = 0.02 vs LR).

#### Restoration of endothelial syndecan-1 by FFP

**Figure 6:**
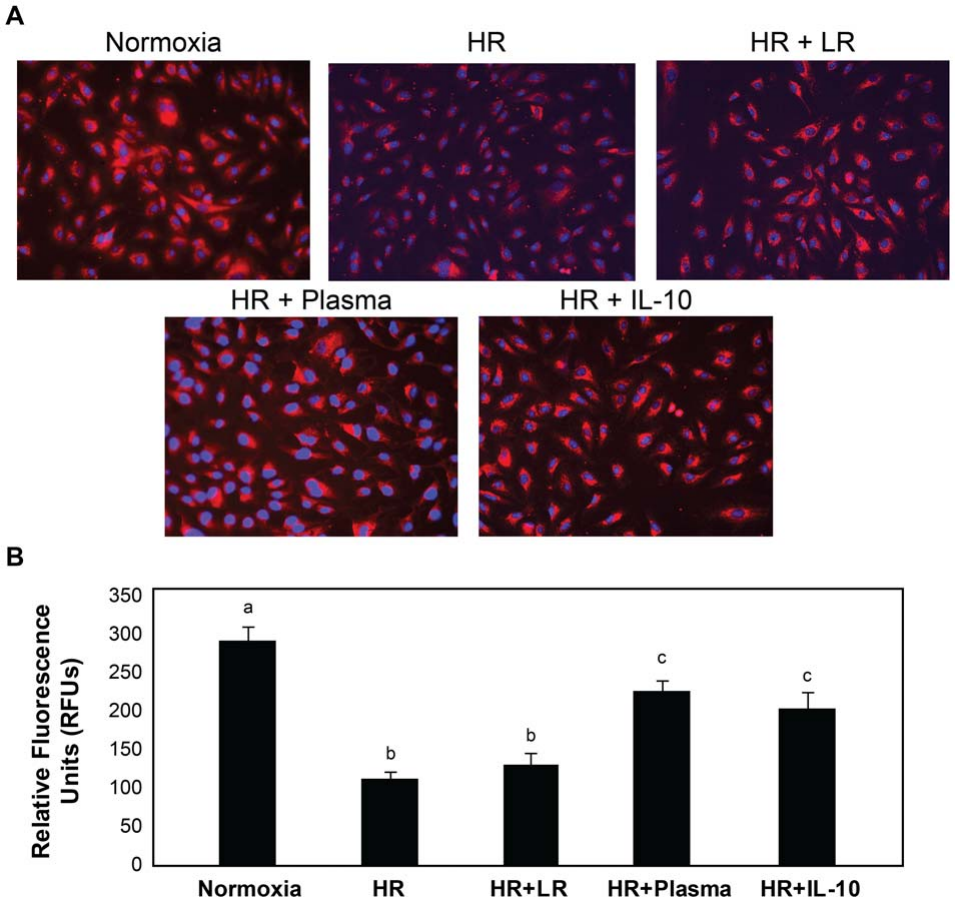
Cell surface syndecan-1 is enhanced by FFP in an in vitro model of endothelial injury. HUVEC's were stimulated with IL-1β and subjected to hypoxia for four hours followed by rexoygenation and incubation for an additional four hours in media alone (hypoxia/reoxygenation, HR), 5% lactated Ringers (LR) or5% FFP and compared to normoxia. A. Cell were labeled with antisyndecan-1 antibodies (magnification = original×200) then B. Images obtained using an Olympus 1×71 microscope with SimplePCI6 software. Original image magnification, ×20. The relative fluorescence intensity was quantified using Image J software (NIH) and reported as relative fluorescence units (RFU). Results are presented as means ± SEM, n = 6 images/group. Means notated with letters indicate statistical differences between groups. Immunostaining revealed that cell surface syndecan-1 is expressed abundantly in normoxia, significantly reduced after HR and LR, but partially restored by FFP.

Endothelial syndecan-1immunostaining was reduced after endothelial injury (114±9 RFU) and resuscitation with LR (132±15) but increased by FFP (228±13) (Figure 6A and B). We have shown similar findings of syndecan-1 preservation by FFP after *in vitro* treatment of cells with heparanase, a syndecan-1 shedding enhancer [23] and in animals after hemorrhagic shock [17]. Importantly, changes in endothelial permeability correlated with syndecan-1 shedding. These data suggest that FFP based resuscitation hastens syndecan-1 restoration compared to LR and may explain the protective properties of FFP in reversing endothelial cell hyperpermeability after shock.

## Discussion

To our knowledge, this is the first report in hemorrhagic shock patients of syndecan-1 shedding and the first correlation between syndecan-1 shedding and inflammatory cytokines. Other investigators have examined shedding of syndecan-1 after sepsis, surgery, and ischemia/reperfusion, however, syndecan-1 levels in the current study markedly exceed those previously reported [24], [25]. After initial shock resuscitation, levels of syndecan-1 dropped significantly when compared to pre-resuscitation but remained elevated above baseline. Its dramatic rise suggests a systemic insult to the endothelium extending beyond the mechanical site of physical injury. Though only a trend, syndecan-1 levels were numerically higher in nonsurvivors than survivors in this small pilot study. The low level of shed syndecan-1 in healthy donors is due to constitutively shed syndecan-1 as part of normal cell syndecan turnover [26].

**Figure 7:**
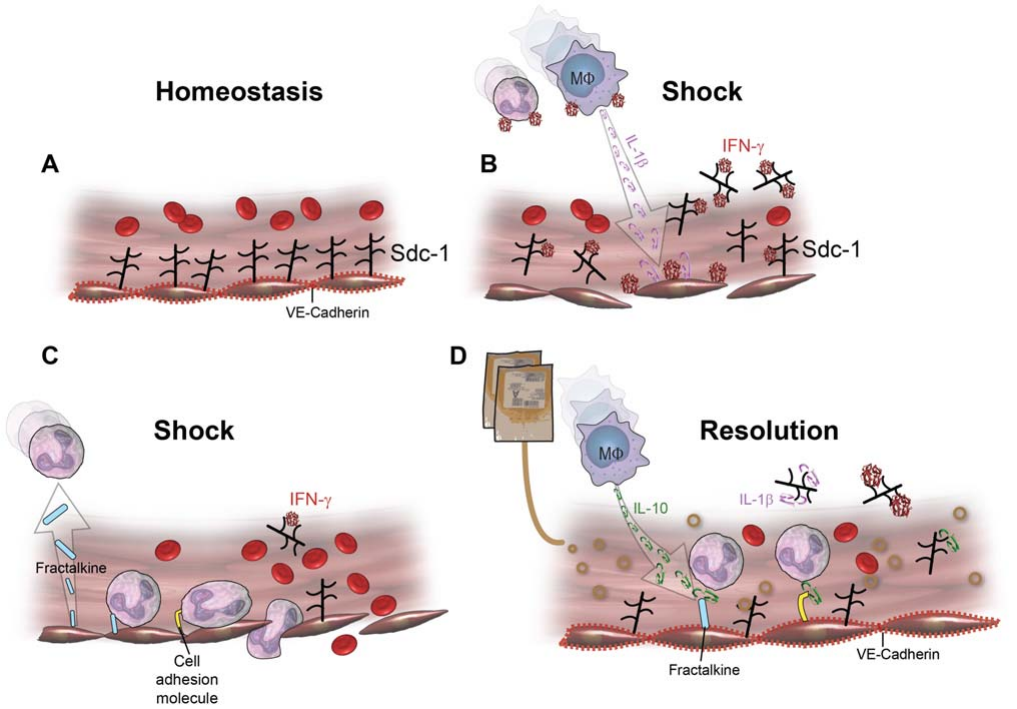
Proposed model of syndecan-1 interaction with inflammatory cytokines after shock and resuscitation with FFP. (A) Syndecan-1 (sdc-1) is a major constituent of the protective glycocalyx found on the surface of endothelial cells (ECs). (B) During hemorrhagic shock, syndecan-1 is shed from the EC surface, exposing the underlying endothelium to pro-inflammatory cytokines. IFN-γ binds the heparan sulfate chains located on syndecan-1 and activates ECs which attracts neutrophils and macrophages to the site of injury. Macrophages further stimulate ECs by secreting IL-1β. (C) Activated ECs secrete fractalkine, a neutrophil chemoattractant, and express cell adhesion molecules on the endothelial cell surface, facilitating pathologic leukocyte-endothelial cell interactions. (D) FFP acts to store vascular integrity. VE cadherins form an intact endothelium. IL-10 counteracts inflammation by decreasing expression of cell adhesion molecules. Shed syndecan-1 facilitates resolution of inflammation by removal of the pro-inflammatory cytokines, IL-1β, IFN-γ, and fractalkine.

Cytokines are mediators of vascular damage after injury, and *in vitro* as well as *in vivo* experiments document pathways for their response to injury [27], [28]. Consistent with previous studies, we found a temporal relationship between cytokine production and time of injury [19], [20], [29]. We report a novel correlation between inflammatory cytokines and syndecan-1 shedding, thus establishing an association between hemorrhagic shock, inflammation, and endothelial cell surface damage. IFN-γ, fractalkine (CX3CL1), and IL-1β, were negatively correlated with plasma syndecan-1 while IL-10 was positively correlated. IL-1β, IL-10 and IFN-γ have been linked to hemorrhagic shock [30]–[33], but there are no reports linking fractalkine. The association between pro- and anti- inflammatory cytokines and syndecan-1 after hemorrhagic shock suggests a more multifaceted role for syndecan-1 in endothelial injury, including resolution of inflammation (Figure 7). IFN-γ is a pleiotropic pro-inflammatory cytokine that binds to heparan sulfate, a glycosaminoglycan found at the endothelial cell surface, and inhibits its biological activity [34]. In addition, IFN-γ activates endothelial cells to express fractalkine, which has the unique role of being a chemo-attractant to adhesion molecule [35], [36]. Soluble fractalkine potently attracts macrophages and T cells, while the membrane bound form facilitates adhesion of leukocytes, which become activated to secrete more IFN-γ [35]. The recruited macrophages then secrete IL-1β, which induces expression of cell adhesion molecules on the endothelial cell surface through the NF-κB pathway to facilitate transendothelial migration of leukocytes to the site of injury/infection [37]. Together, the three negatively correlated cytokines are all pro-inflammatory and serve to either recruit leukocytes to the endothelium or inhibit syndecan-1 biologic activity. IL-10 was the only positively correlated cytokine, as well as the only anti-inflammatory cytokine found to correlate with syndecan-1. IL-10 is another pleiotropic cytokine that attracts macrophages but acts to down regulate the inflammatory process [38]. More importantly, IL-10 can block NF-κB activity thereby decreasing the expression of cell adhesion molecules on the endothelial cell surface and cease leukocyte transmigration.

Shedding has been implicated as both an injurious and protective mechanism in response to cell stress [39], [40]. The role of syndecan-1 shedding in resolution of inflammation was demonstrated in a mouse model of inflammation by Park and colleagues [41]. They demonstrated that syndecan-1 shedding participated in the removal of sequestered CXC chemokines and resolution of neutrophil accumulation. Furthermore, administration of exogenous heparan sulfate diminished the accumulation of pro-inflammatory chemokines and neutrophils. All of these data support the idea that syndecan-1 may not only be a marker of injury, but also plays a major role in the active resolution of inflammation. Our data add another dimension to this function in that syndecan-1 shedding may also dampen inflammation by regulating the expression of the anti-inflammatory cytokine, IL-10.

The precise mechanism by which FFP mitigates syndecan shedding is unknown but the subject of investigation in our labs. Ectodomain shedding is regulated by multiple signaling pathways converging on a diverse group of proteases, referred to as sheddase, with the metalloproteases being the largest group. We have demonstrated that matrix metalloproteases play an important role in ischemia-reperfusion-induced shock [42]. We hypothesize that matrix metalloproteases are found in hemorrhagic shock plasma and serve as effective sheddases of syndecan-1 and that FFP will inhibit or neutralize sheddases. Alternatively/in addition, our preliminary data supports the hypothesis that FFP restores cell surface syndecan after hemorrhagic shock by initially mobilizing an intracellular pool of preformed syndecan [17]. Re-surfaced syndecan then reconstructs the protective network of proteoglycans to re-establish an effective endothelial barrier and mitigate hyperpermeability.

This pilot study has several important limitations. We examined only a snapshot of the inflammatory process and the microvasculature during the acute resuscitation phase of injury. Studies looking at a longer time course and more frequent sampling in injured patients will give us more insight into the complex functions of syndecan-1 after traumatic injury and hemorrhagic shock. It is possible that the clearance of shed syndecan-1 after resuscitation may be a dilutional effect rather than a true decrease in shedding. However, this same pattern of clearing was also noted by Rehm et al in patients undergoing elective aortic aneurysm repair, at shorter time periods that precluded significant resuscitation [25]. They demonstrated clearance of shed syndecan-1 within 30 minutes of aortic unclamping, thus we did not anticipate delayed clearance after hemorrhagic shock and measured only one post resuscitation syndecan level.

Inflammatory cytokine production after shock can alter endothelial cell interactions leading to breakdown of the endothelial integrity [43]. We utilized an *in vitro* model of endothelial injury to simulate hemorrhagic shock based upon cytokines that correlated with syndecan-1 shedding.. Though correlations were low in this small human pilot study, we verified the findings in our in vitro model of shock. IL-1B worsened endothelial integrity while IL-10 significantly improvedendothelial integrity (data not shown). Further studies with a larger sample size are clearly needed. As has been suggested with sepsis, stabilization and repair of the endothelium may prove to be a therapeutic target after hemorrhagic shock [10], [43]. London et al recently demonstrated that targeting the vascular response of cytokines after severe infections by activation of Slit 2, an endothelium-specific Robo-4-dependent signaling pathway, mitigated hyperpermeability and lessened mortality in mouse models of infection [43]. When cells were treated with Slit2, VE-cadherin increased and permeability decreased. We showed that VE-cadherin was increased and permeability decreased by FFP, suggesting that it may in fact assist in restoring endothelial junctional integrity. FFP was also able to lessen the negative effects of shock on cellular ultrastructure as shown by atomic force microscopy which visually confirmed repair of endothelial junctions. Importantly, these findings were associated with maintenance of syndecan-1 expression. Robo4 is required for the effect of Slit2 on VE-cadherin-mediated vascular barrier function, as shown by London, and there is some evidence, at least in neural cells, that syndecans may be involved in the function of Robo 4 [44], [45].

In summary, we have demonstrated in a small pilot study of severely injured patients with a marked inflammatory cytokine response that shedding of syndecan-1 occurs after hemorrhagic shock and decreases with resuscitation. *In vitro* studies suggest that FFP resuscitation is associated with enhanced cell surface syndecan -1expression and may be beneficial after shock in part due to its ability to restore endothelial junction integrity and permeability. Understanding the complex pathways leading to hemorrhagic shock-induced endothelial dysfunction and subsequent repair are key to developing novel therapies that target endothelium after severe injury.

## Materials and Methods

### Human Study

#### Ethics Statement

This specific study was approved by The University of Texas Health Science Center at Houston (UTHSCH) Committee for the Protection of Human Subjects as waiver of consent.

#### Study Design

This prospective, observational nonrandomized pilot study was conducted from January to June 2009 in the Emergency Department (ED) and Shock Trauma Intensive Care Unit (STICU) at Memorial Hermann Hospital, Houston, TX, a busy Level I trauma center.

Patients were entered into the study after initial evaluation in the emergency department and subsequent admission to the STICU. Included patients met criteria for our shock resuscitation protocol, a standardized decision making algorithm that uses bedside computerized decision support [46]. Criteria for shock resuscitation for this study were defined as: emergency department systolic blood pressure <90 mmHg, and/or base deficit ≥6 mEq/ml and a blood transfusion. Hemorrhagic shock patients were resuscitated with the early use of blood products in a 1∶1∶1 ratio of packed red cells, fresh frozen plasma, and platelets starting in the emergency department with early activation of a massive transfusion protocol when appropriate.

Baseline body core temperature, arterial blood gas, and other standard clinical laboratory blood chemistry analyses (ie, electrolyte, glucose concentration, hemoglobin concentration), and coagulation profile (prothrombin time, international normalized ratio [INR], platelet count, and partial thromboplastin time) were obtained on admission and repeated upon arrival to the shock trauma ICU. An aliquot of excess volume collected in a standard plasma EDTA tube that was not used for standard clinical laboratory analyses was transferred to 3-mL sample tubes. The tubes were centrifuged at 1,000 *g* for 15 minutes at 4°C and plasma was immediately collected and stored at −80°C in 400 µL aliquots until analysis. Additional data characterizing patient demographics, shock resuscitation, and ICU course were obtained using our Trauma Research Database and Trauma Registry. This database is maintained with approval of the UTHSCH Committee for the Protection of Human Subjects. Plasma from screened, type specific, single, healthy normal donors was obtained from Gulf Coast Regional Blood Center, Clinical Trials Laboratory, Houston, Texas, and stored at −80°C until analysis.

#### Plasma syndecan-1 and cytokine measurements

Plasma syndecan-1 levels were assessed using enzyme linked immunoabsorbent assay (ELISA) according to manufacturer's instructions (Diaclone; Besancon, France). Plasma was warmed in a 37°C water bath for up to 1 hour with periodic mixing until completely thawed. Once thawed, plasma was centrifuged at 1,000 *g* at 4°C for 15 minutes and 1 ml was sterilely aliquoted into 1.5 ml microcentrifuge tubes and stored at −80°C.

Cytokine concentration analyses were performed using a 39plex suspension immunoassay according to manufacturer's instructions (Multiplex; Millipore). Concentrations of 39 cytokines were measured in each sample. All cytokine measurements were performed on the same day, with ED and STICU samples from an individual patient run on the same plate.

#### Statistical Analysis

Sample time points were referenced as admission to the ED (pre-resuscitation) and ICU arrival (post-resuscitation). We focused on these two time points as data previously described by our group demonstrated dramatic differences in cytokine concentrations detectable 4 to 8 hours after injury [20]. More specifically, we were interested in the inflammatory changes, if any, induced by our standardized shock resuscitation protocol. Demographic data was entered and blood collected on 32 patients in the pre-resuscitation group. Syndecan data was omitted from analysis in one patient with a value more than 2 standard deviations above the others. There were 28 shock patients in the pre-resuscitation group for plasma cytokine analysis due to limited sample quantity in this initial pilot study. The post-resuscitation group for syndecan-1 and cytokine analysis contained 24 patients: 5 patients died prior to obtaining the post-resuscitation blood sample and in 3 patients excess blood was not available for analysis. Forty healthy donor samples obtained from commercially available plasma samples were included in both assays for comparison. Syndecan-1 levels were analyzed using a two sided paired t-test. Since cytokine concentrations show significant variations among subjects, the non-parametric Wilcoxon rank-sum test was used. In both the analyses of syndecan-1 levels and cytokine concentrations, unpaired tests were used to compare cytokine levels in normal donors versus pre-resuscitation and, normal donors versus post-resuscitation while paired tests were used to compare, and pre-resuscitation versus post-resuscitation groups. Seventeen cytokines had a large number of out of range (OOR) measurements (defined as >15%), mostly out of range below (OOR<) with only two cytokines out of range above (OOR>). OOR< was replaced with 0.001 and OOR> with 1 [20]. IL-4 was excluded from the analysis because all measurements were OOR<. Comparisons were considered as statistically significant using Bonferroni adjustment for multiple testing.

To study the correlation between each cytokine and syndecan-1, we fitted a simple linear model for each cytokine, using the generalized estimating equation (GEE) with independent working correlation matrix to account for correlations between the same patients' measurements at pre-resuscitation and post-resuscitation time points. The Bonferroni correction was used to control for multiple comparisons.

### *In vitro* studies

#### Cell culture model

The human study revealed an association of inflammatory cytokines and syndecan-1 in hemorrhagic shock patients resuscitated with both standard of care lactated Ringers and the early use of FFP (Table 1). Our hypothesis was that hemorrhagic shock would disrupt endothelial integrity by promoting syndecan-1 shedding from the endothelial cell surface and that resuscitation with FFP would restore vascular integrity. To test this hypothesis in an *in vitro* model of shock, human umbilical vascular endothelial cells (HUVECs) were exposed to hypoxia/reoxygenation plus IL-1β, as it was a pro-inflammatory cytokine that correlated with syndecan shedding.

HUVEC's were grown on eight well Lab-Tek permanox chamber slides (Nalge Nunc Int., Rochester, NY) coated with 200 µl of collagen (50 µg/ml) per well and incubated overnight. Three ×10^4^ endothelial cells were seeded per well and cells were incubated for 48 hours or until confluency was reached. At the time of experimentation, cells were placed in 1% hypoxia plus 20 ng/ml IL-1β for four hours. Upon reoxygenation, media was replaced with either 5% lactated Ringers or 5% FFP for an additional four hours [18].

#### VE-Cadherin and Syndecan-1 Immunofluorescence

Cells were seeded into 8 well-CultureSlide (BD Falcon™) at 3×10^4^ endothelial cells per well and treated as described as above. At end of reoxygenation, media was removed and cells fixed in 100% cold methanol on ice for 20 minutes followed by paraformaldehyde fixation. Cells were blocked with 2.5% goat serum and 2%BSA in TBS for 1 hour at room temperature, then incubated with 1∶200 primary antibodies for Syndecan-1 (Santa Cruz 12765) or VE-cadherin (Cell Signaling 2500) in 2% BSA in TBST overnight at 4°C. Secondary antibodies ( Alexa Fluor 488, goat anti-rabbit IgG, 1∶1000, or Alexa Fluor 568 goat anti-mouse IgG, 1∶500) were applied in 2% BSA in TBST for 1 hour at room temperature. Cell nucleolus was stained with DAPI for 5 min. Slides were cover-slipped using Fluoromount-G and images were captured with 1×71 inverted microscope (Olympus; Center Valley, Penn). The relative fluorescence intensity was quantified using Image J software (NIH) and reported as relative fluorescence units (RFU).

#### Atomic Force Microscopy

HUVECs were cultured on 8 well chamber slides and treatments were administered as described above. After treatment, the cells were fixed for 20 min in formalin at room temperature. Atomic Force Microscopy was conducted using a BioScope II™ Controller (Veeco Metrology Inc., Santa Barbara, CA) integrated with a Nikon TE2000 inverted optical microscope. AFM studies were accomplished on ‘never dried’ fixed cells to investigate the topographical properties of the cell membrane. Liquid scanning was performed in PBS 1× in contact mode using MLCT cantilevers (f*o* = 4–10 kHz, k = 0.01 N/m) operated to 0.25 Hz for cell description. The image analysis was performed with the Research NanoScope software version 7.30.

#### *In vitro* permeability

24 well transwell cell culture inserts (BD Falcon, 353495) were coated with 1 µg/ml of collagen per insert and incubated overnight at 4°C or at room temperature for 2 hours. One mL of Endothelial Growth Media (Lonza, CC-3162) was added to each well of a 24 well plate. The transwell inserts were placed into the 24 well plate and 4×10^4^ endothelial cells were added to each transwell. The plate was incubated under normal conditions until confluency was reached, usually 48 hours, then fluoresceine-isothiocyanate [FITC] labeled dextran 1∶50 was added for 2 hours. After 15 minutes, 100 µl was taken from the bottom of each well and read immediately at ∼495/520 nm (Biotek Synergy 2). Permeability was assayed using in vitro Vascular Permeability Assay Kit (Millipore, ECM640). Twenty four well transwell inserts were coated with 1 µg/ml of collagen/ insert and incubated at room temperature for 1 hour. 500 ul of Endothelial Growth Media (Lonza, CC-3162) was added to each plate then HUVEC's seeded at 2×105 cells per insert and cultured until formation of integrated cell monolayer. Cells were treated as described above. At end of reoxygenation FITC-Dextran (150 µl) was added to the upper chamber and fluorescent measurements determined using excitation/emission wavelengths of 485 nm/530 nm.

#### Statistical analysis

Data is reported as mean ± SEM. Results were analyzed by one-way ANOVA with Tukey post hoc tests. In figures, means notated with letters indicate statistical differences between groups.

## Supporting Information

Table S1 Median cytokine concentrations.
(DOC)
