# Supplementary material for: Modulation of Syndecan-1 Shedding after Hemorrhagic Shock and Resuscitation
Source: PLoS One. 2011 Aug 19;6(8):e23530. doi: 10.1371/journal.pone.0023530 (PMC3158765; doi:10.1371/journal.pone.0023530)
Supplement: Table S1 — Median cytokine concentrations. (DOC) [file pone.0023530.s001.doc]

| Table 1. Median cytokine concentrations (pg/mL) for normal donor and shock patients | | | |
| --- | --- | --- | --- |
|  | Normal Donors | Pre-resuscitation | Post-resuscitation |
| Cytokine | Median(IQR) | Median(IQR) | Median(IQR) |
| EGF | 24.3(4.1,41) | 10(0,60.6) | 7.2(0,45.8) |
| Eotaxin | 44.2(35.6,56.6) | 50.5(38.9,88.2) | 50(34.1,101.2) |
| FGF-2 | 117.1(93.2,137) | 84.8(66.8,114.4) | 64(52,118.6) |
| FLT-3L | 19.8(0,33.1) | 0(0,16.1) | 0(0,19.2) |
| Fractalkine*^cd^* | 297.6(244.8,379.5) | 124.4(80.7,183.8) | 153.7(108.4,270.4) |
| G-CSF*^d^* | 135.5(112.5,153.4) | 142.7(82.9,1329.9) | 681.4(262,1974.1) |
| GM-CSF | 24.8(15.9,33.5) | 25.6(12.9,37.7) | 30.5(15.7,46.3) |
| GRO*^cd^* | 402.5(329.6,499.3) | 630.7(432.2,961.9) | 616.4(435.8,1029.1) |
| IFN-α2*^c^* | 44.8(32.4,52.8) | 20.6(8.7,39.3) | 25.1(17.9,52.8) |
| IFN-γ*^cd^* | 33.6(20.5,48.8) | 9.6(4.7,13.9) | 9.3(5.3,22) |
| IL-1α | 39.1(20.8,55.2) | 6.4(0.1,27.1) | 10.3(0,52.6) |
| IL-1β*^c^* | 20.8(15.2,26.7) | 6.7(4.6,9.7) | 7(4.3,22.1) |
| IL-1ra | 64.7(42.4,86.6) | 28.7(12,223.2) | 240.9(60.2,1371.1) |
| IL-2*^cd^* | 4.1(3.1,6.3) | 0.4(0,2.2) | 0.6(0,3.7) |
| IL-3*^cd^* | 4.6(2.8,5.8) | 0.8(0.1,2.8) | 0.9(0.1,3.5) |
| IL-4 | 0(0,0) | 0(0,0) | 0(0,0) |
| IL-5 | 0(0,0.3) | 0(0,0) | 0(0,0) |
| IL-6*^cd^* | 1(0,3.8) | 192.4(81.8,812.8) | 326.7(150.9,729) |
| IL-7*^c^* | 17.1(11.9,23.8) | 4.6(0,12.8) | 6.1(4.2,24.1) |
| IL-8*^cde^* | 4.3(2.6,7.3) | 29.1(9.8,47.6) | 72.3(29.2,238.3) |
| IL-9 | 0(0,2.6) | 1.4(0,3.3) | 3.1(0,9) |
| IL-10*^cd^* | 6.9(4.6,11) | 74(40.7,172.4) | 49(25.7,106.6) |
| IL-12.p40 | 38(8.8,133.2) | 48.4(0,128) | 59.6(11.7,101) |
| IL-12.p70*^c^* | 12(7,16.6) | 3.5(1,10.6) | 4.3(1.5,13.6) |
| IL-13*^cd^* | 8(3.9,11.5) | 0(0,0) | 0(0,0) |
| IL-15 | 2.6(0.9,4.7) | 1.1(0,2.5) | 2.4(1.1,5) |
| IL-17*^c^* | 4.8(2.3,6.9) | 0.5(0,1.5) | 0.8(0,3.6) |
| IP-10 | 303.4(269.7,402.5) | 478.8(279.1,682.3) | 400.6(216.5,723.1) |
| MCP-1*^cd^* | 181.6(152.1,216.8) | 821.1(526,1638.7) | 1026.7(456.8,3192) |
| MCP-3 | 36.5(24,48.7) | 15.3(11.1,22.3) | 15(11.4,28.4) |
| MDC | 808.7(674,976.7) | 787(559.5,948.8) | 854(748.1,1044) |
| MIP-1α | 28.3(19.5,44.4) | 18.8(2.5,26.2) | 22.3(12.1,33.8) |
| MIP-1β | 62.2(47.7,77.6) | 57.5(42.4,89.3) | 68(40.8,105) |
| sCD40L*^cd^* | 902(680,1290) | 2969(2106,4677) | 2052(1645,2724) |
| sIL-2ra | 0(0,4.5) | 0(0,20.1) | 6.3(0,55.6) |
| TGF-α | 4.4(0.4,7.5) | 4.9(2.3,11.9) | 2.1(0.2,7) |
| TNF-α | 5.6(4,8.8) | 7.5(4.7,11.8) | 8.2(5.5,12.7) |
| TNF-β*^c^* | 26.8(13.8,42.5) | 0.5(0,10.8) | 8.5(3.2,21.1) |
| VEGF | 0(0,93) | 31.4(0,65.4) | 35(10.6,99.7) |

IQR = inter-quartile range (1^st^ quartile, 3^rd^ quartile).

*c, d, e*: Statistically significant difference between ND and pre-resuscitation (c) , ND and post-resuscitation (d), pre- and post-resuscitation (e).

EGF, epidermal growth factor; FGF-2, fibroblast growth factor-2; Flt-3L, FMS-like tyrosine kinase-3 Ligand; G-CSF, granulocyte colony-stimulating factor; GM-CSF, granulocyte-macrophage colony-stimulating factor; GRO, growth regulated protein; IFN, interferon; IL, interleukin; IP-10, inhibitor protein-10; MCP, monocyte chemotactic protein; MDC, macrophage-derived chemokine; MIP, macrophage inflammatory protein; sCD40L, soluble cluster of differentiation 40 ligand; sIL-2Rα, soluble interleukin 2 receptor α; TGF- α, transforming growth factor- α; TNF, tumor necrosis factor; VEGF, vascular endothelial growth factor.
